# Applications of dynamic gratings in neuro-ophthalmic disorders

**DOI:** 10.3389/fmed.2026.1892604

**Published:** 2026-07-20

**Authors:** Xinzuo Zhou, Yuexin Wang

**Affiliations:** 1 Department of Ophthalmology, Peking University Third Hospital, Beijing, China; 2 Peking University Health Science Center, Beijing, China

**Keywords:** amblyopia, diabetic retinopathy, dynamic gratings, glaucoma, multiple sclerosis, Parkinson's disease, schizophrenia

## Abstract

Traditional static vision-based assessment evaluate only stationary, spatial modulated resolution, leaving early and pathway-specific visual impairments undetected. Dynamic gratings are periodically modulating bright-dark stripe stimuli with precisely controllable spatiotemporal parameters that preferentially engage magnocellular and parvocellular visual pathways. Despite this, dynamic grating has not been applied in routine practice due to a lack of standardized protocols, normative databases, and clearly defined clinical indications. This is a narrative review which comprehensively summarizes the motion patterns of dynamic gratings and their clinical utility in neuro-ophthalmic disorders. The five primary motion patterns include phase-alternating oscillation, drift, translation, appearance-disappearance, and rapid serial visual presentation. Through parameter modulation of dynamic gratings, psychophysical and neurophysiological responses are collected, to provide multidimensional evidence for disease mechanism elucidation, diagnosis, and treatment evaluation. These applications are reviewed spanning a spectrum of ophthalmic and neuro-ophthalmic conditions, including glaucoma, amblyopia, strabismus, Graves' ophthalmopathy, diabetic retinopathy, multiple sclerosis, Parkinson's disease, and schizophrenia. The current research provided a foundation for the application of dynamic gratings in clinical ophthalmology. Potential future research should focus on establishing disease-specfic parameter sets and developing an integrated platform to enable standardized clinical implementation.

## 1 Introduction

Traditional static visual acuity testing evaluates only high-contrast spatial resolution under stationary conditions, whereas real-world visual stimuli involve numerous moving targets. Assessment based solely on static optotypes cannot fully reflect visual function or detect early, subtle, or pathway-specific visual impairments (1). Therefore, it is essential to develop assessment tools that simulate natural visual environments and evaluate the visual system's capacity to process dynamic stimuli, in order to better understand the mechanisms of visual dysfunction and enable early diagnosis of ocular diseases.

Among the various dynamic visual stimuli, dynamic gratings have emerged as a central tool for investigating visual system function, owing to their precisely controllable parameters and clearly defined neural mechanisms (2–4). There is currently no consensus on the definition of dynamic gratings in visual science. Based on previous literature, dynamic gratings are defined as visual stimuli of bright-dark stripes that exhibit periodic or sequential temporal changes. Spatial frequency describes stripe density, defined as the number of cycles per degree of visual angle. Temporal frequency describes the rate of temporal change in the stripes, with its precise definition depending on the motion pattern. Schade first introduced dynamic gratings to visual science (5). By using stationary and drifting sinusoidal gratings and manipulating their spatial frequency, this study measured the sine-wave response of the visual system, establishing a theoretical foundation for future investigations.

Systematic modulation of grating parameters allows preferential engagement of distinct neuronal subtypes in the visual pathway (2, 3, 6). Low spatial frequency, high temporal frequency stimuli preferentially activate the magnocellular (M) pathway, which is sensitive to motion and global spatial information. While high spatial frequency, low temporal frequency stimuli preferentially activate the parvocellular (P) pathway, which is responsible for fine form and color processing. This “pathway-biased stimulation” capability offers a powerful non-invasive approach for functionally dissecting disease-induced damage to the hierarchical visual pathways extending from the retina to the cortex (7–11). Building on this foundation, by modulating the parameters of dynamic gratings and concurrently collecting psychophysical response parameters [e.g., contrast sensitivity (11–13), motion direction discrimination threshold (14, 15), detection and discrimination thresholds (16, 17), flicker fusion frequency (15)] and neurophysiological signal parameters [e.g., visual evoked potential (VEP) (7, 18, 19), electroretinogram (ERG) (20–22), electroencephalogram (EEG) (23), functional magnetic resonance imaging (fMRI) (23)], multi-dimensional objective evidence can be generated for disease mechanism elucidation, diagnosis, and treatment evaluation, encompassing glaucoma (12, 24, 25), amblyopia (26, 27), strabismus (28, 29), Graves' ophthalmopathy (19, 30), diabetic retinopathy (31, 32), multiple sclerosis (33), Parkinson's disease (10) and schizophrenia (11, 34).

The purpose of this review is to systematically synthesize the patterns of dynamic gratings and their clinical applications in ophthalmology, and to assess their utility in uncovering disease mechanisms, enabling early diagnosis, informing differential diagnosis, and evaluating treatment outcomes.

## 2 The patterns of dynamic gratings

**Figure 1:**
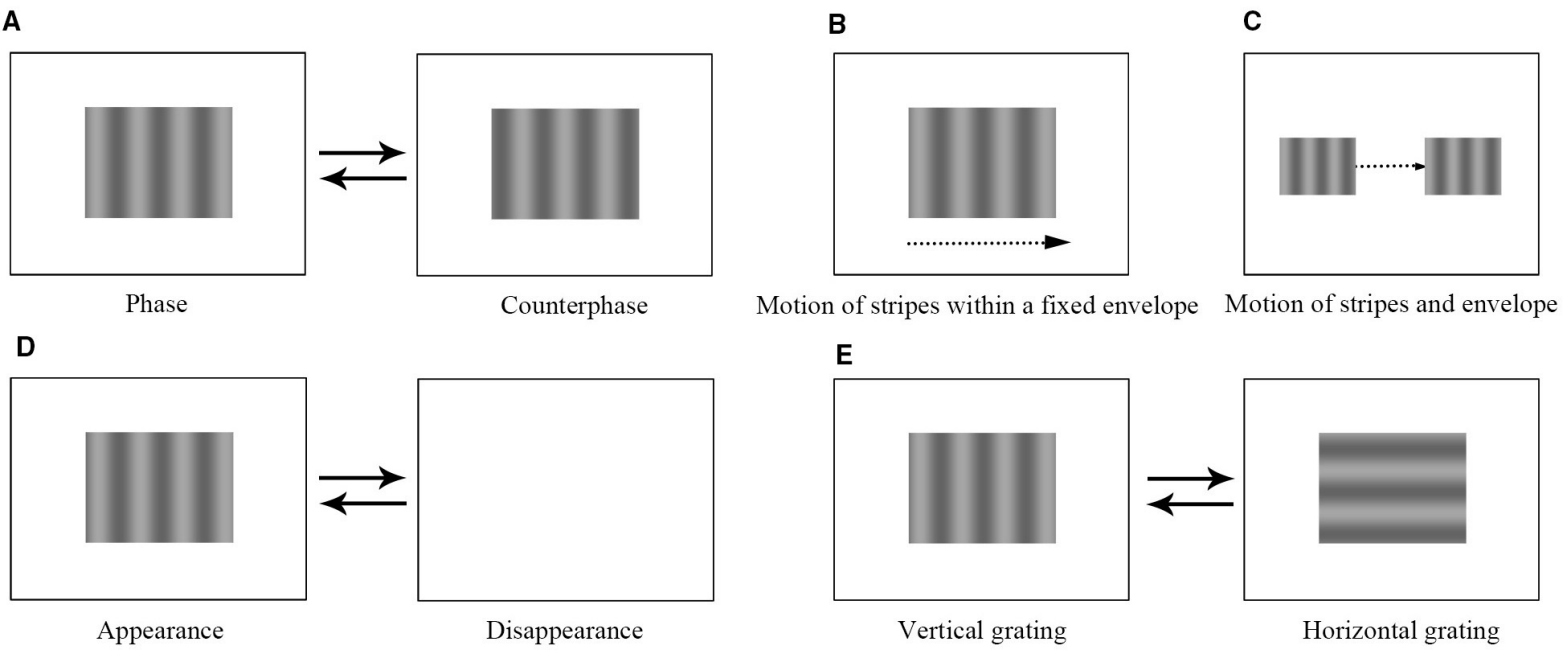
Schematic of the five motion patterns of dynamic gratings. **(A)** Phase-alternating oscillatory grating (counterphase); **(B)** Drifting grating; **(C)** Translational grating; **(D)** Appearance-disappearance grating; **(E)** Rapid serial visual presentation (RSVP) grating. The solid arrow indicates a periodic shift between two gratings. The dotted arrow indicates the motion.

There are mainly five patterns of dynamic gratings: phase-alternating oscillation, drift, translation, appearance-disappearance, and rapid serial visual presentation, as shown in Figure 1 and Table 1.

**Table 1:** Basic dynamic grating paradigms and parameters.

Paradigm	Spatial frequency	Temporal frequency or speed	Contrast	Phase	Direction of motion	Envelope movement	Typical clinical applications
Phase-alternating	Cycles per degree of visual angle	Phase oscillation cycles per second	Variable	180° or 90°	N/A	Envelope fixed	Visual evoked potential, electroretinogram, contrast sensitivity, frequency-doubling perimetry
Drifting	Cycles per degree of visual angle	Cycles of stripes drifting past a fixed point per second	Variable	N/A	Perpendicular to stripe orientation	Envelope fixed	Motion perception, optokinetic nystagmus, visual evoked potential
Translational	Cycles per degree of visual angle	Rate of spatial displacement or oscillation cycles per second	Variable	N/A	Perpendicular to stripe orientation or back-and-forth along the motion axis	Envelope moves with stripes	OPTOKINETIC nystagmus, visual evoked potential
Appearance-disappearance	Cycles per degree of visual angle	Appearance-disappearance cycles per second	variable	N/A	N/A	Envelope fixed	Visual evoked potential
Rapid serial visual presentation	Cycles per degree of visual angle	Number of grating patterns presented per second	Variable	N/A	N/A	Envelope fixed	Orientation tuning

### 2.1 Phase-alternating oscillation

Phase-alternating oscillatory gratings are dynamic gratings in which the phase alternates periodically between an initial value and a peak value, resulting in either reversal or jitter, of the bright-dark stripes, while the overall envelope remains stationary at a fixed location (Figure 1A) (2–4, 29, 35). The temporal frequency describes the speed of phase oscillation, defined as the number of phase oscillation cycles completed per second. One oscillation cycle corresponds to a complete process in which the grating phase starts from an initial value, increases to a peak, and then returns to the initial value. The phase indicates the position of its bright and dark stripes. The amplitude of phase oscillation can be adjusted based on experimental needs. Two amplitudes are commonly used. An amplitude of 180° produces a reversal of the bright-dark stripe positions, which is perceived as flickering and is therefore also called counterphase (2–4). An amplitude of 90° produces positional jittering of the stripes (29, 35).

### 2.2 Drift

Drifting gratings are dynamic gratings in which the stripes move continuously in a particular direction within a fixed spatial envelope, whereas the overall outline remains stationary without spatial displacement (Figure 1B) (11, 17, 29, 31, 36–39). The temporal frequency describes the rate at which luminance changes over time, defined as the number of cycles the stripes drift spatially per second. One cycle corresponds to a complete bright-dark stripe pair sweeping past a fixed location along the drift direction. Consequently, drifting gratings create the visual impression of stripes continuously moving in a particular direction. The drift direction of a grating is orthogonal to its stripe orientation. The higher the temporal frequency, the faster the stripes drift. Nevertheless, the spatial envelope of a drifting grating is fixed, meaning the physical presentation region of the grating on the display does not change. This constitutes the fundamental difference in motion pattern between drifting gratings and the translational gratings discussed below.

Because stripes can only provide motion signals that are perpendicular to their orientation (40, 41), the drift direction of a grating must be orthogonal to its stripe orientation. Specifically, for a grating with vertical stripes, the drift direction is horizontal (17). For a grating with stripes oriented at 45°, the drift direction is 135° (17).

### 2.3 Translation

Translational gratings are dynamic gratings in which the spatial envelope and the internal stripes, as an integrated whole, shift continuously and synchronously in spatial position (Figure 1C) (10, 42–44). The displacement speed refers to the rate of change of their spatial position over time, defined as the degrees of visual angle per second that the grating pattern translates across space. This constitutes the fundamental difference in motion pattern between translational gratings and the drifting gratings described earlier. When a grating pattern translates back and forth within a fixed spatial extent, it exhibits the motion pattern known as oscillation. The temporal frequency of an oscillatory translational grating is defined as the number of oscillation cycles the grating pattern completes per second, where one oscillation cycle corresponds to one full reciprocating translation. Oscillatory translational gratings can be considered a special spatial modulation of translational gratings (10, 43, 44).

### 2.4 Appearance-disappearance

Appearance-disappearance gratings are dynamic gratings in which the grating pattern alternates with a blank background at a constant frequency (Figure 1D) (8). The temporal frequency describes the rate of change of the grating pattern over time, defined as the number of appearance-disappearance cycles completed per second, where one cycle consists of the presentation of the grating pattern and the subsequent blank interval.

### 2.5 Rapid serial visual presentation

Rapid serial visual presentation (RSVP) gratings are dynamic gratings in which a series of gratings with different patterns are presented consecutively at a constant frequency (Figure 1E) (45). The temporal frequency describes the rate of change of the grating pattern over time, defined as the number of grating patterns presented per second; consequently, the duration of each grating pattern equals the reciprocal of the temporal frequency. It is important to note that RSVP gratings differ from the appearance-disappearance gratings described above in that the former lacks a blank interval, presenting instead a continuous stream of grating patterns that vary in parameters (45).

## 3 Applications of dynamic gratings in ophthalmology and vision-threatened neural disorders

### 3.1 Glaucoma

Dynamic gratings are valuable for evaluating optic nerve injury in glaucoma. Prior research has demonstrated that glaucoma patients experience substantial loss of nerve fibers prior to the development of visual field defects (46). The histological study conducted by Quigley et al. revealed that large-diameter optic nerve fibers degenerate more rapidly than their small-diameter counterparts, and that some glaucoma patients show marked fiber loss even when visual fields are still normal (47). These observations provided the basis for later research on the selective vulnerability of optic nerve fiber subtypes in glaucoma. Using a 1.2 c/d, 8 Hz counterphase grating, Atkin et al. observed that patients with primary open-angle glaucoma (POAG) exhibited significantly reduced dynamic contrast sensitivity compared to healthy controls, leading them to hypothesize that glaucoma may cause preferential injury to Y-type retinal ganglion cells (12). Subsequently, Atkin et al. employed a 2.3 c/d, 1 Hz counterphase grating to elicit pattern visual evoked potential (PVEP) from patients with glaucoma and ocular hypertension (OHT) (7). They found that both groups demonstrated delayed PVEP P100 latencies, indicating abnormal processing of low spatial frequency information by their visual systems and suggesting dysfunction of the M pathway.

Horn et al. reported that high temporal frequency counterphase gratings were strongly correlated with glaucomatous optic nerve damage, yielding correlation coefficients of *r* = 0.80 with mean sensitivity and *r* = 0.59 with neuronal rim area, values that were significantly higher than those obtained with static gratings (48). Using drifting gratings, Turano et al. characterized the temporal filtering properties of the visual motion system and demonstrated that glaucoma predominantly compromises the processing of high temporal frequency motion (36). In the study by Korth et al., translational grating stimuli sensitive to the M pathway were applied while recording VEP (42). The results showed a significant decrease in N200 amplitude in glaucoma patients, and the extent of this decrease was correlated with visual field defects, indicating that motion-evoked VEP may be a useful objective marker of M pathway function.

Nevertheless, not all studies support selective magnocellular damage. Using drifting gratings to assess motion sensitivity, Willis and Anderson found no significant differences between glaucoma patients and healthy controls across all testing conditions (16). Ansari et al. reported that, when using counterphase gratings, the contrast sensitivity loss in early-stage glaucoma patients in the central visual field lacked spatiotemporal frequency specificity (25). Klein et al. employed counterphase gratings to compare dynamic contrast sensitivity in patients with cataract alone vs. those with both cataract and glaucoma (49). They proposed that the functional overlap between the M and P pathways might preclude psychophysical experiments from detecting pathway-specific damage to the M system (50). Moreover, drawing on a hypothesis put forward by Johnson (51), Klein et al. suggested that visual function loss in glaucoma could also be attributable to altered sampling properties or reduced redundancy among different optic nerve fiber subtypes (49).

Beyond its role in mechanisms, dynamic gratings have also been applied to glaucoma diagnosis. Atkin et al. used counterphase gratings to show that both open-angle glaucoma patients (OAG) and OHT individuals had prolonged P100 latencies relative to normal controls, with a greater delay in the glaucoma group, thereby offering initial support for electrophysiological approaches in early glaucoma detection (7). Maddess et al. advanced this technology by recording multiregion pattern electroretinograms (PERGs) using counterphase gratings in normal subjects, glaucoma suspects, and diagnosed glaucoma patients (21). The resulting diagnostic model achieved remarkably high sensitivity and specificity. Vaegan et al. further demonstrated that steady-state visual evoked potential (ssVEP) elicited by counterphase gratings had a higher detection rate for glaucoma than psychophysical measures, indicating that electrophysiological metrics may be more advantageous for early glaucoma diagnosis (18).

The electrophysiological studies discussed above focus on objective measures of neural conduction along the visual pathway, whereas psychophysical tests exploiting the frequency doubling (FD) illusion are more concerned with global perceptual function. Using counterphase gratings as FD stimuli, Johnson and Samuels reported that a 16 smaller stimulus patterns achieved the best diagnostic performance, with an AUROC of 0.965, 100% specificity, and 93% sensitivity (52). Based on this work, Sponsel et al. developed an experimental frequency doubling perimetry (FDP)-based classification system for glaucomatous visual field defects (24). The FDP grading showed strong agreement with Humphrey perimetry grading but required substantially less testing time, thus greatly improving diagnostic efficiency. Maddess and Severt further optimized the screening parameters for FD stimulation (53). By selecting appropriate counterphase grating parameters for different regions of the visual field, they increased sensitivity from 80 to 86.7% and specificity from 84.6 to 90%, thereby improving the accuracy of glaucoma screening.

However, as research has progressed, the specificity of FD stimuli for probing the M pathway has been called into question. White et al. recorded neural responses from macaque M retinal ganglion cells to counterphase gratings and found that the nonlinear responses of these cells failed to produce a FD effect (54). This prompted researchers to introduce chromatic dimensions into dynamic gratings to better assess visual pathways and enhance diagnostic accuracy. Antón et al. developed an ATD system using counterphase gratings with achromatic (A), red-green (T), and blue-yellow (D) modulation, and found that low spatial frequency chromatic stimuli were most effective for detecting glaucomatous damage (55). With RG-0.5/2 and BY-0.5/2 stimuli, pattern standard deviation achieved sensitivities of 89.3 and 82.1%, respectively, at 85% specificity. Wen et al. achieved preferential pathway engagement by saturating the P pathway with red-green counterphase gratings while simultaneously stimulating the M pathway with black-white drifting gratings, allowing early diagnosis of functional impairment in preperimetric glaucoma (56). They reported that in the peripheral location the 25-Hz condition of the new paradigm yielded an AUROC of 0.957, significantly outperforming the FD paradigm (AUROC = 0.761).

The aforementioned diagnostic approaches depend on the active participation of the subjects. For patients with cognitive impairment or poor compliance, the development of fully objective screening measures is essential. Maddess et al. departed from conventional paradigms by recording optokinetic nystagmus (OKN) responses to drifting gratings as well as to gratings that combined drifting with counterphase (57). The resulting diagnostic model achieved high accuracy in classifying normal individuals and glaucoma suspects.

In summary, dynamic gratings have furnished crucial evidence for unraveling the mechanisms underlying glaucomatous optic nerve injury and have concurrently enhanced the performance of early glaucoma screening. As such, they represent a vital link between research into the pathogenesis of glaucoma and its objective clinical evaluation.

### 3.2 Amblyopia and binocular visual dysfunction

Dynamic gratings play an essential role in uncovering the mechanisms underlying visual dysfunction and spatiotemporal deficits in amblyopia. Manny and Levi employed counterphase gratings and reported that amblyopic patients showed markedly reduced flicker sensitivity at medium-to-high spatial frequencies combined with low temporal frequencies (13). In contrast, Bradley and Freeman found virtually no difference in flicker sensitivity between the amblyopic and fellow eyes in these two patient groups when using counterphase gratings (58). They proposed that the flicker sensitivity loss documented in earlier studies was attributable to stimulus size rather than to a genuine temporal visual deficit. Levi and Harwerth used counterphase gratings and demonstrated that patients with severe form deprivation could not reach pattern detection threshold at spatial frequencies above 0.5 c/d, perceiving only flicker (26). Hess et al. investigated spatiotemporal contrast sensitivity in individuals with congenital monocular cataract using both counterphase and drifting gratings (59). Their findings corroborated the conclusions of Levi and Harwerth (26).

Dynamic gratings also serve as an essential tool for investigating the neuropathogenesis of amblyopia. In 1974, Ikeda and Wright hypothesized that amblyopia results from functional degradation of the sustained pathway caused by the absence of clearly focused macular images during early visual development (60). Levi and Harwerth provided psychophysical evidence supporting this hypothesis using counterphase gratings (27). Their research showed that patients with strabismic and anisometropic amblyopia exhibited more severe impairment of dynamic contrast sensitivity at high spatial frequencies and low temporal frequencies, whereas their sensitivity was relatively preserved at low spatial frequencies and high temporal frequencies. The results support the neural mechanism of impaired P pathway function and preserved M pathway function in amblyopic patients. Subsequently, Levi and Harwerth provided electrophysiological evidence for P pathway impairment using appearance-disappearance gratings in VEP recordings (8). Anatomical and physiological studies have shown that the P pathway supplies substantial excitatory input to the superficial layers of V1 (61), where neurons exhibit strong neural selectivity of orientation (62). However, whether and how the neural selectivity of orientation is altered in amblyopia remains debated (45). Building on this, Zhu et al. used RSVP gratings to thoroughly characterize orientation tuning in amblyopia, revealing a multi-tiered impairment involving weakened geniculocortical feedforward input, abnormal intracortical lateral interactions, and delayed downstream decision-making stages (45). Focusing on high spatial frequency deficits in strabismic amblyopia, primarily mediated by the P pathway (8, 27, 60), Demanins et al. used counterphase and drifting gratings to test patients with strabismic amblyopia and identified three distinct patterns of neural deficit: spatial undersampling within a regular array, spatial undersampling within a irregular array, and neural pooling (17). This finding highlights the inherent heterogeneity of the neural mechanism underlying strabismic amblyopia.

Binocular asymmetry of optical or muscular origin during childhood disrupts the normal development of binocular vision (23). Regarding binocular interactions, Apkarian et al. investigated binocular VEPs in patients with monocular strabismic amblyopia using counterphase gratings (63). They observed binocular interaction patterns similar to those of normal controls, indicating that binocular interaction function is not lost in strabismic amblyopia. Lygo et al. used counterphase gratings to collect multimodal data via fMRI and EEG, revealing asymmetrical interocular suppression in amblyopia (23). Regarding binocular fusion, Zhang et al. employed drifting gratings and found that the perceived direction of binocular fusion motion in amblyopic patients was biased toward the fellow eye (14). Conversely, Kosovicheva et al. reported that binocular fusion function is not entirely compromised in amblyopia (15). Using dichoptically presented counterphase gratings with different contrasts and phase delays, they demonstrated that both amblyopic patients and normal controls showed broad flicker fusion temporal tuning, with no significant difference in accuracy.

Directional selectivity in motion perception is a fundamental attribute of the visual system, relying on nonlinear integration mechanisms within cortical direction-selective pathways (62). Patients with early-life binocular vision abnormalities, such as those with infantile esotropia, exhibit various direction biases in oculomotor, perceptual, and cortical evoked potential responses, including nasotemporal asymmetry (29). Kommerell et al., Anteby et al. and Brosnahan et al. collectively explored the multi-level manifestations of nasotemporal asymmetry in infantile strabismus (29, 43, 44). Using oscillatory translating gratings to record OKN responses and motion visual evoked potentials (MVEPs), Kommerell et al. proposed that MVEP asymmetry might partially result from the shift of the retinal image caused by nystagmus (44). In contrast, Anteby et al. refuted this hypothesis (43). By employing horizontally translating and oscillatory translating gratings, they demonstrated that MVEP asymmetry is neither an artifact of nystagmus nor a secondary adaptation but rather a primary deficit in binocular visual cortex circuits. Brosnahan et al. systematically assessed nasotemporal bias across three levels, oculomotor, perceptual and cortical, using drifting gratings and 90° phase-alternating oscillatory gratings (29). They found biases at all three levels, with greater severity associated with earlier age of onset, suggesting parallel damage to these pathways during a shared critical period. Shea et al. further examined the neural mechanisms underlying nasotemporal asymmetry in infantile esotropia (35). Using 90° phase-alternating oscillatory and counterphase gratings, they provided evidence that nasotemporal asymmetry originates from a nonlinear imbalance in direction-selective pathways. Because oscillatory stimulation can elicit an F1 component reflecting this directional imbalance, it may serve as a sensitive tool for early detection of direction-selective pathway dysfunction. Garbutt et al. extended the investigation to the vertical dimension (28). Their study with drifting gratings further confirmed the presence of nasotemporal asymmetry in horizontal oculomotor responses in strabismic patients. Additionally, it revealed enhanced horizontal oculomotor crosstalk when these patients responded to vertical motion stimuli. This finding suggests that early binocular visual loss may induce abnormal coupling and compensation between different directional motion pathways.

Dynamic gratings have not only advanced our understanding of amblyopic mechanisms but also opened new avenues for optimizing treatment strategies. On the optimization of traditional therapy timing, Birch and Stager used phase-reversing and static square-wave gratings in their study (64). They found that contrast sensitivity deficits were comparable between children who underwent surgery for monocular congenital cataract within 8 weeks of birth and those with bilateral congenital cataract. However, when form deprivation extended to 12–30 weeks, monocular cataract children exhibited significantly greater contrast sensitivity loss, providing strong evidence for early surgical intervention in form-deprivation amblyopia. On the development of novel therapies, Kämpf et al. introduced pleoptic training with dynamic grating into practice using a home-based computer game system superimposed with drifting gratings (65). They found that this novel therapy was effective in patients with occlusion-resistant refractory amblyopia, achieving faster visual improvement, suggesting its role as a supplementary treatment. In a subsequent crossover study, Kämpf et al. confirmed that patients who received dynamic grating training first showed better visual outcomes (39). This suggests that dynamic grating training with occlusion should be used early, not just for late-stage refractory cases.

In summary, dynamic gratings have been instrumental in advancing the understanding of strabismus and amblyopia. They have uncovered the multi-layered mechanisms of neural injury in these disorders and highlighted the heterogeneity of their neural deficits. Moreover, dynamic gratings have offered valuable insights for refining therapeutic approaches.

### 3.3 Graves' ophthalmopathy

The early clinical features of Graves‘ ophthalmopathy are nonspecific, and conventional ophthalmic examinations typically yield normal results, making accurate differentiation from other optic nerve disorders crucial (19). Bobak et al. employed counterphase gratings to record both transient and steady-state VEPs (19). They found that patients with Graves' ophthalmopathy exhibited normal P1 latency but abnormal second harmonic amplitude, which differed from that observed in patients with retrobulbar optic neuritis or pseudotumor cerebri. These findings indicate that combined transient and steady-state VEPs can aid in distinguishing compressive optic neuropathy from other forms of optic nerve injury.

As Graves' ophthalmopathy progresses, enlargement of the extraocular muscles can compress the optic nerve at the orbital apex, leading to dysthyroid optic neuropathy (DON). To avert irreversible visual loss, Marco et al. explored the use of dynamic gratings for early detection of DON (30). Using counterphase gratings, they measured contrast sensitivity in three groups: patients with uncomplicated Graves' ophthalmopathy, those with OHT accompanying Graves' ophthalmopathy, and patients with DON. The findings indicated that low-spatial-frequency dynamic gratings were more effective at identifying patients with DON in clinical practice.

In summary, dynamic gratings offer important value for differential diagnosis in Graves' ophthalmology and enable early detection of DON, thus helping in the prevention of irreversible visual impairment.

### 3.4 Diabetic retinopathy

Diabetic retinopathy (DR) is the most common chronic complication of diabetes mellitus and a leading cause of irreversible vision loss and blindness in adults (32). Consequently, accurate evaluation and long-term monitoring of DR are essential. Ghirlanda et al. proposed that the second harmonic component of the PERG originates from retinal ganglion cells and the inner retina, indicating that ganglion cell dysfunction emerges early in diabetes (32). Falsini et al. employed counterphase dynamic gratings to record steady-state PERG in patients with type 1 diabetes mellitus (T1DM) (22). Their results showed that patients with disease onset after age 13 had lower PERG second harmonic amplitudes than those with onset before age 13, while disease duration showed no correlation with second harmonic amplitude. This finding contradicts common expectations. Researchers hypothesized that pubertal hormonal changes and elevated levels of secondary growth factors may increase retinal vulnerability to diabetes. In contrast, Caputo et al. reported opposing results (66). They found a significant negative correlation between second harmonic amplitude and duration of T1DM, but no correlation with age of onset. These findings support the use of steady-state PERG for early detection and monitoring of T1DM progression. By comparison, Zhang et al. focused on type 2 diabetes mellitus (T2DM) (31). Using drifting gratings, they found that reduced motion sensitivity to high spatial frequency second-order stimuli was significantly associated with DR risk in T2DM.

In summary, dynamic gratings are effective for evaluating early neural injury in DR, tracking disease progression, and detecting retinal ganglion cell dysfunction. As such, they provide valuable functional measures for objective assessment and long-term surveillance of the disease.

### 3.5 Multiple sclerosis and optic neuritis

Multiple sclerosis (MS) is a chronic autoimmune disorder of the central nervous system. According to current research, nearly three-quarters of MS patients show detectable optic nerve abnormalities, and approximately half of them will experience optic neuritis (67).

Regarding pathological mechanisms, MacCana et al. employed counterphase gratings to measure pattern and motion detection thresholds across several ophthalmic conditions (9). In MS patients, the two thresholds were reduced to different extents, indicating that the disease may cause differential damage to the P and M pathways. Medjbeur and Tulunay-Keesey reinforced this finding using counterphase gratings, showing that different patients exhibited distinct patterns of spatiotemporal frequency loss (33). Rucker et al. extended these observations with oculomotor experiments, recording ocular following response (OFR) to drifting gratings in MS patients (37). They found no significant difference in contrast–OFR latency curves between the affected and fellow eyes at low spatial frequencies, but a decreased slope in the affected eye at high spatial frequencies. This suggests a relative preservation of the M pathway and more severe P pathway damage. However, the study included only a single patient, which limits the generalizability of its findings.

For precise diagnosis, Camisa et al. employed counterphase gratings to record VEPs from MS patients (68). They demonstrated that VEP latencies were dependent on grating orientation, and that using gratings with any two orientations could enhance diagnostic accuracy and reduce false negatives. For differential diagnosis, Hess and Plant focused on distinguishing optic neuritis from compressive and ischemic optic neuropathies (69). Using counterphase gratings, they found that the low-spatial-frequency contrast sensitivity deficit in optic neuritis was temporal frequency-dependent. This was a characteristic not observed in the other two conditions, which offered differential diagnostic value. For early detection, Medjbeur and Tulunay-Keesey found that although the contralateral unaffected eye had normal Snellen acuity, it showed contrast sensitivity deficits (33). This finding indicates that contrast sensitivity measurement with dynamic gratings is a sensitive marker of subclinical impairment in MS.

In summary, dynamic gratings have uncovered the mechanisms underlying visual pathway injury in optic neuritis associated with MS (9, 33, 37). They have also provided critical insights for diagnosis and differential diagnosis, and detection of subclinical damage, thus improving the sensitivity of clinical evaluation (33, 68, 69).

### 3.6 Parkinson's disease

Parkinson's disease (PD) is the second most common neurodegenerative disease, marked by resting tremor. Accumulating evidence suggests that a subset of patients exhibit visual impairments (70).

Using counterphase gratings, Stanzione et al. found that oral administration of haloperidol to healthy individuals significantly prolonged the PERG b-wave latency, whereas VEP latencies remained unchanged (71). This finding suggests that dopaminergic blockade influences the regulation of retinal horizontal cells. Because PD involves damage to dopaminergic neurons, Stanzione et al. administered the selective D2 receptor antagonist sulpiride to healthy subjects to mimic the disease (72). They observed a marked reduction in P50 amplitude and a significant delay in second harmonic latency following injection. This pattern was consistent with previously reported PERG alterations in PD patients. These findings suggest that dysfunction of widely distributed D2 receptors in the retina may contribute to the visual symptoms of PD. Tagliati et al. also employed counterphase gratings to record PERGs (20). They found a significant negative correlation between the PERG tuning ratio and the clinical stage of PD, particularly in patients not receiving levodopa. This result indicates that PERG could serve as a useful marker for disease monitoring and treatment evaluation in PD.

Color discrimination abnormalities in PD were investigated by Haug et al. and Sartucci et al. (10, 73). Haug et al. employed chromatic gratings to evaluate both static and dynamic visual functions in PD patients (10). They found that the blue-yellow pathway was severely affected, the luminance pathway was moderately affected, whereas the red-green pathway remained largely intact. This pattern suggests that the underlying pathology in PD involves the early stages of the retinal parvocellular-blue-yellow pathway dedicated to color processing. Sartucci et al. reinforced this conclusion by using red-green, blue-yellow, and yellow-black counterphase gratings to record PERGs (73). They observed a significant PERG latency delay exclusively for the blue-yellow grating, thus providing electrophysiological evidence supporting the aforementioned findings.

In summary, dynamic gratings provide a unique tool for evaluating both retinal dopaminergic function and visual function in PD, owing to their temporal modulation characteristics (10, 20, 71–73).

### 3.7 Schizophrenia spectrum disorders

Schizophrenia is a multifactorial neurodevelopmental disorder. Many studies have shown that visual function impairment is also one of its important characteristics (74).

Slaghuis tested 15 patients with predominantly negative symptoms and 15 with predominantly positive symptoms using drifting gratings (11). The results showed that the positive-symptom group had M pathway impairment with relative sparing of the P pathway, while the negative-symptom group exhibited dysfunction in both pathways. Slaghuis and Thompson subsequently employed a focal-peripheral stimulus paradigm to examine the far-out jerk effect in positive- and negative-symptom patients, and their results supported the notion that different visual pathway deficits exist between the two schizophrenia subtypes (75). In contrast, Cadenhead et al. used counterphase luminance gratings and red-green gratings and found no significant group differences in dynamic contrast sensitivity between schizophrenia patients and those with schizotypal personality disorder (SPD) (76). The authors therefore proposed that the schizophrenia spectrum is associated with generalized deficits in visual information processing. Such deficits cannot be attributed to selective damage of the M or P pathway alone.

With respect to dopaminergic function in the schizophrenia spectrum, Kéri et al. employed counterphase gratings to examine the impact of antipsychotic drugs on dynamic contrast sensitivity (77). They found that higher doses of dopamine receptor blockers were correlated with more severe contrast sensitivity deficits. This result indicates that hypodopaminergic function may underlie visual impairments in schizophrenia. Chen et al. further compared typical and atypical antipsychotics using drifting gratings (34). Their results revealed that, relative to healthy controls, the typical antipsychotic group exhibited decreased contrast sensitivity, the atypical antipsychotic group showed no significant difference, and the unmedicated group demonstrated increased contrast sensitivity. The researchers interpreted these findings as follows: unmedicated schizophrenia patients are in a hyperdopaminergic state, which enhances the distinction between center and surround retinal neural activity and improves contrast sensitivity; typical and atypical antipsychotics differ in their D2 receptor blocking profiles, leading to distinct effects on contrast sensitivity. Kent et al. extended this line of inquiry to SPD, another condition within the schizophrenia spectrum (78). Using counterphase gratings to measure dynamic contrast sensitivity in unmedicated SPD patients, they found that all SPD patients showed reduced contrast sensitivity compared with healthy controls. This finding suggests that untreated SPD patients are in a hypodopaminergic state.

In summary, dynamic gratings, owing to their precise modulation of spatiotemporal frequency, have facilitated research on visual pathway impairments in schizophrenia spectrum disorders as well as investigations of dopaminergic function (11, 34, 75–78).

### 3.8 Other disorders of the central nervous system

Beyond the conditions discussed above, dynamic gratings have also been applied to study the mechanisms of other central nervous system disorders. Battista et al. employed drifting grating either with or without a surrounding annular grating to evaluate the magnitude of visual surround suppression in migraine patients (38). Their findings supported the view that migraine involves an imbalance in the inhibitory and excitatory visual processes. Porrello and Falsini used counterphase gratings to record PERGs from the hemiretina (79). They found that post-geniculate lesions can induce trans-synaptic retrograde dysfunction, and that this pathological process exhibits selectivity for P pathway subtypes.

## 4 Clinical translation of dynamic grating-based assessments

A critical question in the clinical adoption of dynamic grating-based testing is how these novel assessments compare with existing diagnostic tools. Dynamic grating-based testing devices offer several advantages over conventional visual function assessments. Compared with standard automated perimetry (SAP), PERG, FDP, and Pulsar perimetry substantially reduce test duration (24, 80–83), show greater sensitivity to early functional deficits, and are better tolerated by patients with cognitive impairment, while maintaining good diagnostic accuracy (21, 24, 80, 81, 83, 84). Relative to OCT, these functional tests correlate reasonably well with structural parameters (55, 81, 85, 86), and can reveal subclinical dysfunction in regions deemed normal on OCT, or detect functional loss beyond what structural imaging alone can explain (55, 86, 87). Nevertheless, the structure–function correlation in glaucoma remains only modest, suggesting that the two approaches capture different aspects of the disease (88). When compared with conventional static contrast sensitivity testing, dynamic grating-based contrast sensitivity and perimetry provide better discriminative ability for early detection and differential diagnosis, with lower variability and higher reliability (30, 85). However, findings from dynamic VEP and static contrast sensitivity are not fully consistent, implying that they may probe distinct functional domains of the visual pathway (89). In VEP testing, checkerboard patterns remain the clinical standard for VEP recording, favored for their robust responses and low intersubject variability. However, they differ fundamentally from sinusoidal gratings in spatial frequency content: the checkerboard contains prominent higher harmonics, which may obscure selective spatial frequency deficits along the visual pathway (90). This distinction is clinically consequential—in Parkinson's disease, for instance, grating stimuli revealed VEP prolongation in 69% of patients, while checkerboard stimulation detected abnormalities in only 8% of the same cohort (91). Collectively, these findings indicate that dynamic grating-based tests should not be viewed as substitutes for conventional methods, but rather as complementary tools. Their combined use enables a more comprehensive characterization of visual dysfunction (21, 30, 80–83, 85, 86, 89, 91), improves the integration of structural and functional information (55, 81, 85, 86, 88), and may aid in differentiating the underlying mechanisms of optic nerve damage (92), ultimately leading to more precise clinical decision-making.

Having established the comparative advantages of dynamic grating-based tools, it is important to identify which clinical scenarios are most likely to benefit from their application. Dynamic grating-based assessment is particularly well suited to glaucoma, as it directly aligns with the disease's underlying pathophysiology. By independently modulating spatial and temporal frequencies and incorporating chromatic contrast, this technique enables targeted evaluation of the magnocellular pathway. Moreover, functional measures derived from dynamic grating testing—including perimetry and psychophysical tasks—exhibit clear structure–function relationships with OCT parameters. The widespread clinical adoption of FDP and Pulsar perimetry further attests to the practicality of this approach. Collectively, these attributes position glaucoma as one of the prime candidates to benefit from dynamic grating-based evaluation.

Beyond identifying the most suitable diseases, it is equally critical to clarify the specific roles that dynamic grating-based tests can play across different stages of clinical management. In ocular and central nervous system disorders, dynamic gratings hold promise as a versatile platform, facilitating early diagnosis, differential diagnosis, treatment evaluation, and disease monitoring, while also offering unique insights into pathophysiological mechanisms.

Finally, to ensure that these promising tools can be reliably translated into routine practice, a clear framework for technical standardization is essential. To facilitate clinical translation and ensure data reliability, the standardization of dynamic grating-based tests should address four key areas. First, hardware calibration must regulate display luminance and contrast, and ensure a stable, sufficiently high refresh rate matched to the temporal frequency of the stimulus. Second, testing conditions should enforce a fixed viewing distance via a chin rest and incorporate real-time fixation monitoring to minimize artifacts from eye movements. Third, testing protocols should define appropriate stimulus configurations for specific diseases—for instance, prioritizing lower spatial frequencies in glaucoma—and employ validated threshold estimation strategies. Finally, interpretation standards require the establishment of age-stratified normative databases and clear reliability criteria to distinguish true functional loss from noise. Implementing these technical requirements will be essential for the widespread and consistent clinical adoption of dynamic grating-based assessments.

However, when using dynamic grating devices, researchers must be aware of the risk of photosensitive epilepsy (PSE) — a condition marked by photoparoxysmal responses (PPR) to visual stimuli such as flicker, high-contrast gratings, moving patterns, and rapidly modulating luminance (93). Different grating motion patterns vary in epileptogenicity; counterphase gratings are more likely to trigger seizures than drifting gratings (94). Pattern parameters also matter: striped gratings are more epileptogenic than checkerboards, especially at spatial frequencies of 1–4 c/d; the risk also increases with rising contrast within the 0.2–0.4 range, and with increasing pattern size. Stripe orientation does not affect risk, and isoluminant red-green gratings are non-epileptogenic (95). Therefore, to minimize seizure provocation, testing parameters should be carefully optimized, and patients should be screened for personal or family history of epilepsy.

## 5 Conclusions

This review systematically manifested patterns of dynamic gratings and their clinical applications in ophthalmology and vision-threatening neurological disorders. By enabling manipulation of spatial and temporal frequency, dynamic gratings provide a versatile platform for selectively probing magnocellular and parvocellular pathway function. At present, dynamic gratings are primarily used with visual electrophysiology and psychophysical paradigms, including motion detection, VEP, ERG, EEG, and fMRI. The key clinical evidence is summarized in Supplementary Table 1. The clinical findings show that dynamic gratings have unique value for mechanism elucidation, early diagnosis, differential diagnosis, and treatment evaluation in ocular and central nervous system disorders. Dynamic gratings can address the limitations of traditional static visual acuity, contrast sensitivity, and perimetry. They thus serve as an important supplement for clinical visual function assessment. However, several issues remain. These include a lack of standardized protocols and unclear clinical indications. There is also the potential risk of inducing PSE. In addition, how dynamic grating measurements relate to other visual function indices remains unclear. Future research should clarify the core value of dynamic gratings in visual function assessment and developing integrated hardware-software platforms that streamline testing. By clarifying the unique value of dynamic grating assessment and fostering methodological standardization, the field can actively move toward the incorporation of these tools into ophthalmic and neuro-ophthalmic care.
