# Supplementary material for: Applications of dynamic gratings in neuro-ophthalmic disorders
Source: Front Med (Lausanne). 2026 Jul 20;13:1892604. doi: 10.3389/fmed.2026.1892604 (PMC13430467; doi:10.3389/fmed.2026.1892604)
Supplement: Supplementary file 1 [file Table_1.docx]

Supplementary Table 1. Clinical applications of dynamic gratings in different disease

| **Disease** | **Study Population** | **Grating Paradigm** | **Key Parameters** | **Outcome Measures** | **Main Findings** | **Clinical Implication** |
| --- | --- | --- | --- | --- | --- | --- |
| **Glaucoma** | POAG, OHT, preperimetric glaucoma, glaucoma suspects | counterphase, drifting, translational | low spatial frequency with high temporal frequency to stimulate M pathway | contrast sensitivity, VEP, PERG, FDP thresholds, OKN | M pathway dysfunction remains debated due to conflicting psychophysical evidence. Dynamic gratings detect functional loss in glaucoma before standard perimetry, particularly via FDP and PERG. Functional deficits correlate with structural optic nerve damage and visual field severity. | mechanism elucidation, early screening, objective assessment, diagnostic adjunct |
| **Amblyopia and Binocular Visual Dysfunction** | anisometropic amblyopia, strabismic amblyopia, form-deprivation amblyopia, infantile esotropia | counterphase, jitter, drifting, translational, oscillatory translational, appearance-disappearance, RSVP | high spatial frequency with low temporal frequency to stimulate P pathway, low spatial frequency with high temporal frequency to stimulate M pathway, variable orientations | contrast sensitivity, orientation tuning, VEP, OKN, binocular fusion | P pathway is predominantly impaired in amblyopia, whereas M pathway remains relatively preserved. Infantile esotropia manifests nasotemporal asymmetry across oculomotor, perceptual, and cortical levels, reflecting early direction-selective pathway deficits. Early surgery and dynamic grating-based training offer therapeutic benefits. | mechanism elucidation, treatment timing, novel therapy |

Supplementary Table 1 (continued)

| **Disease** | **Study Population** | **Grating Paradigm** | **Key Parameters** | **Outcome Measures** | **Main Findings** | **Clinical Implication** |
| --- | --- | --- | --- | --- | --- | --- |
| **Graves’ Ophthalmopathy** | GO without complications, GO with OHT, DON | counterphase | 0.18-15.7 c/d,  0-6.87 Hz | VEP, contrast sensitivity | Dynamic grating VEP can distinguish compressive optic neuropathy from other optic neuropathies. Low-spatial-frequency dynamic gratings effectively identify patients with dysthyroid optic neuropathy in clinical settings. | differential diagnosis, early detection of DON |
| **Diabetic Retinopathy** | T1DM, T2DM | counterphase, drifting | 1.7 c/d, 8Hz, first- and second-order motion | PERG, motion sensitivity | Retinal ganglion cell dysfunction precedes microvasculopathy in diabetic patients. PERG abnormalities correlate with either disease duration or age of onset, though evidence remains conflicting. Reduced motion sensitivity to high-spatial-frequency stimuli predicts diabetic retinopathy in T2DM. | early detection, disease monitoring |

Supplementary Table 1 (continued)

| **Disease** | **Study Population** | **Grating Paradigm** | **Key Parameters** | **Outcome Measures** | **Main Findings** | **Clinical Implication** |
| --- | --- | --- | --- | --- | --- | --- |
| **Multiple Sclerosis and Optic Neuritis** | MS with / without optic neuritis, demyelinating optic neuropathy | counterphase, drifting | low-to-medium spatial frequency, low-to-medium temporal frequency | VEP, contrast sensitivity, motion detection threshold, OFR | Multiple sclerosis causes differential damage to P and M pathways. Combining multiple grating orientations and low-spatial-frequency stimuli aids both diagnosis and differential diagnosis from other optic neuropathies. Subclinical dysfunction is detectable in the contralateral unaffected eye. | mechanism elucidation, differential diagnosis, subclinical detection, diagnostic accuracy |
| **Parkinson’s Disease** | PD (various stages, on / off levodopa), healthy subjects with D2 blockers | counterphase (chromatic) | blue-yellow, red-green and luminance gratings | PERG, contrast sensitivity | Blue-yellow pathway is predominantly affected in Parkinson‘s disease, whereas red-green pathway remains largely intact. D2 receptor blockade reproduces PD-like PERG changes, implicating retinal dopaminergic dysfunction. PERG tuning ratio declines with disease progression and may serve as a treatment-monitoring biomarker. | mechanism elucidation, disease monitoring, treatment evaluation, dopaminergic function assessment |

Supplementary Table 1 (continued)

| **Disease** | **Study Population** | **Grating Paradigm** | **Key Parameters** | **Outcome Measures** | **Main Findings** | **Clinical Implication** |
| --- | --- | --- | --- | --- | --- | --- |
| **Schizophrenia Spectrum Disorders** | schizophrenia (positive / negative symptoms), SPD, medicated/unmedicated patients | counterphase, drifting | low spatial frequency with high temporal frequency to stimulate M pathway, high spatial frequency with low temporal frequency to stimulate P pathway | contrast sensitivity, motion detection | The pattern of visual pathway damage differs between positive- and negative-symptom schizophrenia patients. Dopaminergic status varies across medication states and across schizophrenia spectrum disorders, as reflected by contrast sensitivity changes. | mechanism elucidation, dopaminergic function assessment, antipsychotic effect evaluation |

**Note**: POAG = primary open-angle glaucoma; OHT = ocular hypertension; M pathway = magnocellular pathway; P pathway = parvocellular pathway; VEP = visual evoked potential; PERG = pattern electroretinograms; FDP = frequency doubling perimetry; OKN = optokinetic nystagmus; RSVP = rapid serial visual presentation; GO = Graves’ ophthalmopathy; DON = dysthyroid optic neuropathy; T1DM = type 1 diabetes mellitus; T2DM = type 2 diabetes mellitus; MS = multiple sclerosis; OFR = ocular following response; PD = Parkinson’s disease
